# Interleukin-17 induces human alveolar epithelial to mesenchymal cell transition via the TGF-β1 mediated Smad2/3 and ERK1/2 activation

**DOI:** 10.1371/journal.pone.0183972

**Published:** 2017-09-05

**Authors:** Ting Wang, Yuan Liu, Jing-Feng Zou, Zhen-Shun Cheng

**Affiliations:** Department of Respiratory Medicine, Zhongnan Hospital of Wuhan University, Wuhan, China; University of South Alabama Mitchell Cancer Institute, UNITED STATES

## Abstract

Idiopathic pulmonary fibrosis (IPF) is a chronic and usually progressive lung disease and the epithelial-mesenchymal transition (EMT) may play an important role in the pathogenesis of pulmonary fibrosis. IL-17 is a proinflammatory cytokine which promotes EMT profiles in lung inflammatory diseases. In this study, we investigated the effect of IL-17 on EMT in alveolar epithelial cell line A549 and the role of TGFβ1-Smad and ERK signaling pathways in the process. Morphological observation on the cells was performed under inverted microscope. The mRNA and protein expressions of E-cad and α-SMA were detected by quantitative RT-PCR and western blotting. The mRNA and protein expressions of TGF-β1 were analyzed via quantitative RT-PCR and ELISA. Expressions of Smad2/3, p-Smad2/3, ERK1/2, p-ERK1/2 and p-JNK were examined by western blotting. The results indicated that IL-17 can induce A549 cells to undergo morphological changes and phenotypic markers changes, such as down-regulated E-cad expression and up-regulated α-SMA expression. Additionally, IL-17 enhanced TGF-β1 expression and stimulated Smad2/3 and ERK1/2 phosphorylation in A549 cells. However, there were no significant differences in the expression of phosphorylated JNK in A549 cells with or without IL-17 treatment. SB431542 or U0126 treated cells showed inhibited morphological changes and phenotypic markers expression, such as up-regulated E-cad expression and down-regulated α-SMA expression. In summary, our results suggest that IL-17 can induce A549 alveolar epithelial cells to undergo EMT via the TGF-β1 mediated Smad2/3 and ERK1/2 activation.

## Introduction

Idiopathic pulmonary fibrosis (IPF) is a specific form of chronic, progressive fibrosing interstitial pneumonia of unknown cause [1]. Its prognosis is devastating and lung transplantation is the only curative therapy [2]. The pathogenic mechanisms are unclear, but a growing body of evidence indicates that the disease is the result of an abnormal behaviour of the alveolar epithelial cells and the epithelial-mesenchymal transition (EMT) may play an important role in the pathogenesis of pulmonary fibrosis [3]. EMT is a process when epithelial cells gradually transform into mesenchymal-like cells losing their epithelial functionality and characteristics [4]. During this process, epithelial cells lose their characteristic cell-cell adhesion structures, change their polarity and cell-cell adhesion structures, and acquire a mesenchymal phenotype including a morphological transition from a cobblestone-like epithelial phenotype to a spindle-like mesenchymal phenotype, which is accompanied by the markers changes, such as the decreased expression of epithelial markers E-cadherin and the increased expression of mesenchymal markers α-SMA [5].

Previous studies have identified a number of chemokines, cytokines, and growth factors mediating EMT in pulmonary fibrosis, such as TGF-β1 [6] and IL-17 [7], which are essential for the development of pulmonary fibrosis. IL-17 is family of proinflammatory cytokines which is composed of six similar members including IL-17A (the first described IL-17), IL-17B, IL-17C, IL-17D, IL-17E and IL-17F. Although IL-17A is expressed by adaptive- and immune-cell types, including CD8+ T-cells, γδ T-cells, natural killer T-cells and innate lymphoid cells, Th17 cells were thought as a major source of IL-17A [8]. Currently, there is emerging evidence that IL-17 is involved in the pathogenesis of pulmonary fibrosis [7, 9]. Vittal et al [7] found that IL-17-mediated col(V) expression and EMT may occur via TGF-β1-dependent pathways in obliterative bronchiolitis. Moreover, Mi et al [9] found that IL-17A antagonism inhibited chronic inflammation and pulmonary fibrosis in a TGF-β1-dependent manner.

TGF-β1 is a pleiotropic factor that has been indentified as a potent driver of the EMT during embryonic development, wound healing, fibrotic diseases, and cancer pathogenesis [10]. TGF-β1 is known to stimulate the EMT through two main pathways: the canonical Smad-dependent pathway and a non-Smad signaling pathway. Smad family are important intracellular mediators of TGFβ signaling, however, it’s unclear whether they participate in exerting IL-17-induced EMT. Additionally, the activated receptors may also signal through other signal transducers, for example, the mitogen-activated protein kinase (MAPK) pathways, including the extracellular signal regulated kinases (ERKs), c-Jun amino terminal kinase (JNK) and p38 MAPK [11]. Moreover, it is becoming increasingly evident that ERK signaling pathway is implicated in chronic fibroproliferative diseases. For instance, Chen et al [12] found that TGF-β1-mediated renal fibrosis relies on ERK signaling pathways activation. Tan et al [13] suggested that IL-17A-dependent hepatic stellate cell activation and collagen expression through ERK1/2 signaling provide a mechanism of fibrogenesis. For instance, Chen et al [12] found that TGF-β1-mediated renal fibrosis relies on ERK signaling pathways activation. Tan et al [13] suggested that IL-17A-dependent hepatic stellate cell activation and collagen expression through ERK1/2 signaling provide a mechanism of fibrogenesis. However, little is known about the role of ERK pathway in IL-17-induced pulmonary fibrosis.

Taken together, we hypothesized that IL-17 may induce EMT in lung epithelial cells through TGF-β1 mediated Smad or ERK signaling pathway. In this study we aimed to investigate the role of Smad and ERK signaling pathways in EMT induced by IL-17 to identify possible targets for the treatment of pulmonary fibrosis.

## Materials and methods

### Cell culture and preparation

A549, the human alveolar epithelial cell line, was purchased from the the Type Culture Collection of the Chinese Academy of Sciences, Shanghai, China. A549 cells were cultured in RPMI-1640 (Hycolne, Logan, UT, USA) containing 10% heat-inactivated fetal bovine serum (FBS, Sciencell, San Diego, California, USA) and 1% antibiotics (100 u/ml penicillin and 100 μg/ml streptomycin) at 37°C in a humidified atmosphere of 5% CO2 in air. Cell cultures were routinely split when 85–90% confluent.

3 × 10^5^ cells were seeded into 6-well plates in RMPI-1640 containing 10% FBS. To induce EMT, cells were incubated with IL-17 (Peprotech, Rocky Hill, NJ, USA) at a final concentration of 100 ng/ml for 48 h. In the untreated controls RMPI-1640 medium was added. Cells were cultured for up to 48 h. To investigate the potential pathways that might be involved in TGF-β1 signaling with respect to EMT induced by IL-17, cells were preincubated for 1 h with a TGF-β receptor type I kinase inhibitor SB431542 (Selleckchem, Houston, Texas, USA) at 10 μM or an ERK inhibitor U0126 (Selleckchem) at 10 μM before treatment with exogenous IL-17.

### Quantitative RT-PCR

Total RNA was extracted with TRIzol (Invitrogen, Carlsbad, CA, USA) according to the manufacturer’s instructions. RNA was then used to synthesize the cDNA with the Prime Script RT Reagent Kit (TaKaRa, Dalian, China) following the manufacturer’s instructions. The cDNA was used as a template for real-time PCR analysis: reactions were carried out in a CFX96 system (BioRad). The amplification program performed by the manufacturer’s instructions, as follows: 2 min. at 95°C, and then 40 cycles of 5 sec. at 95°C and 30 sec. at 60°C. Relative mRNA levels of the target genes were normalized to GAPDH mRNA expression and analyzed by the 2^-ΔΔCt^ method. The sequences of specific primers were listed as follows: E-cadherin: forward,5’- TTCTTCGGAGGAGAGCGG-3’,reverse,5’-CAATTTCATCGGGATTGGC-3’; α-SMA: forward,5’-CTATGCCTCTGGACGCACAAC-3’,reverse,5’-CCCATCAGGCAACTCGTAACTC-3’;TGF-β1:forward,5’-CAGCAACAATTCCTGGCGATA-3’,reverse,5’-GCTAAGGCGAAAGCCCTCAAT-3’;GAPDH:forward,5’-GGTCGGAGTCAACGGATTTG-3’,reverse,5’-GGAAGATGGTGATGGGATTTC-3’.

### Western blotting analysis

Harvested cells were washed twice with cold PBS and then lysed using RIPA buffer (Beyotime, Shanghai, China) containing a protease inhibitor cocktail (Selleck, Shanghai, China). The lysates were centrifuged for 15 min at 12,000×g at 4°C and the supernatant was collected. The supernatant was boiled for 5 min and then separated by 10% SDS-PAGE gels. The proteins were transferred to a PVDF membrane (Millipore, Bedford, MA, USA), blocked with 5% milk, and incubated overnight with E-cadherin (Abcam, Cambridge, MA, USA), α-SMA (Abcam), TGF-β1 (Abcam), phosphorylated ERK1/2 (Cell Signaling Technology, Boston, USA), ERK1/2 (Cell Signaling Technology), phosphorylated Smad2/3 (Cell Signaling Technology), Smad2/3 (Cell Signaling Technology) and phosphorylated JNK (Cell Signaling Technology). Membrane was then washed three times in TBST and incubated with peroxidase-labelled secondary antibodies (Boster, Inc., Wuhan, China) for 2 h at room temperature. Following three washes in TBST, bands were visualized by western electrochemiluminescence (ECL) kit (Tanon, Shainghai, China) and then exposed to X-ray film. The band densities were quantified using the AlphaEaseFC image analyzer system (Alpha Innotech, Inc., CA, USA) and the results were expressed as ratio of band density to total GAPDH.

### Enzyme-linked immunosorbent assay (ELISA)

The cell culture medium was centrifuged to remove cellular debris and the culture superrnatant was collected. TGF-β1 protein levels were performed with a Human TGF-β1 ELISA Kit (Abcam) following the manufacturer’s instructions.

### Statistical analyses

All data were analyzed by SPSS (version 20.0) and expressed as means ± SEM of at least three independent experiments. One-way ANOVA was used for multiple comparisons. Student’s *t*-test was used for pairwise comparisons between two groups. *P*<0.05 was considered statistically significant.

## Results

### IL-17 induced alveolar epithelial cells to undergo EMT

**Fig 1:**
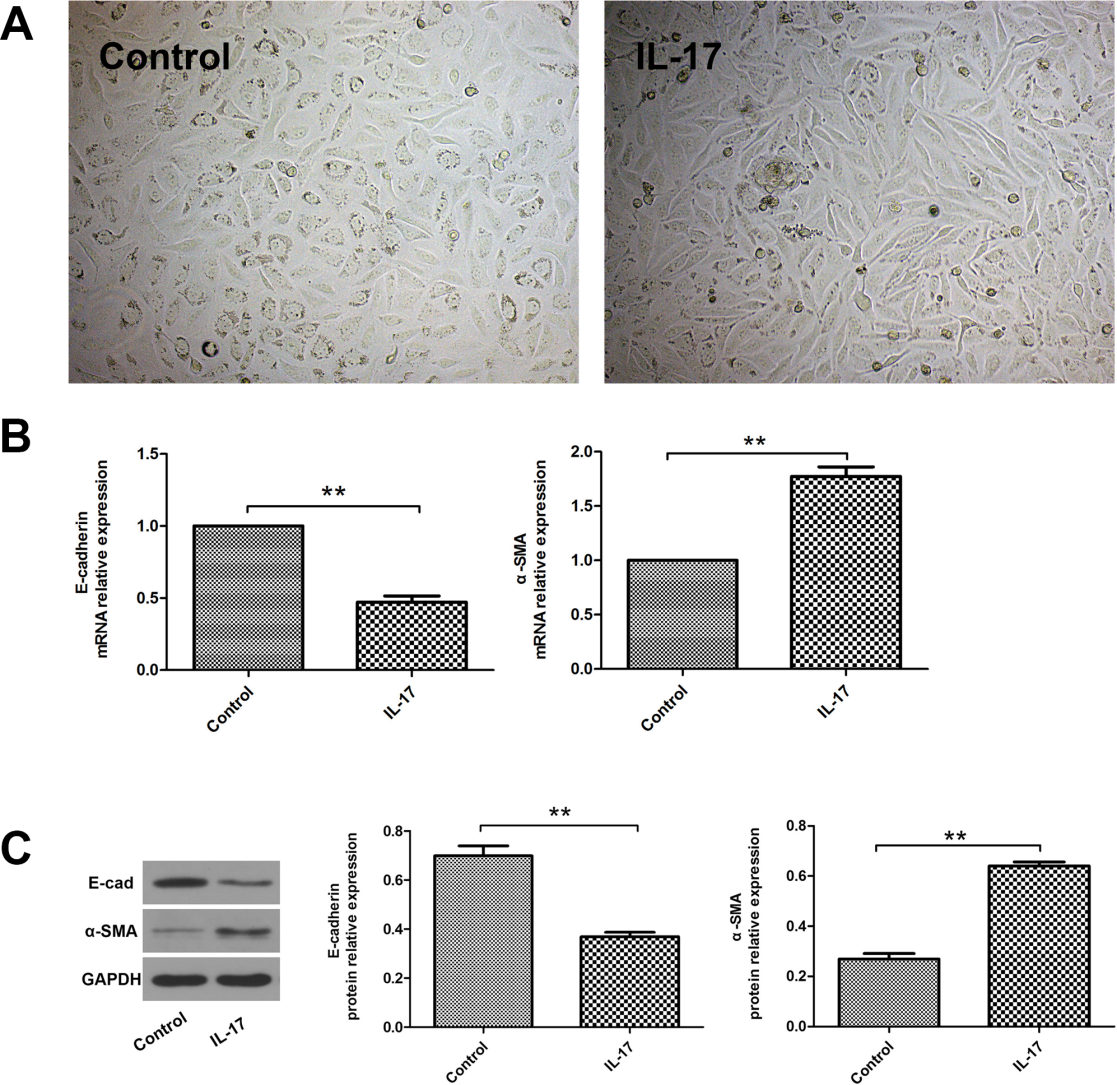
IL-17 induced A549 cells to undergo EMT. The A549 cells were stimulated with IL-17 at a final concentration of 100 ng/ml for 48 h. (A) Phase contrast analysis (magnification ×200) of morphological changes detected in A549 cells. (B) Quantitative RT-PCR analysis of E-cadherin and α-SMA mRNA expressions in A549 cells with or without IL-17 treatment for 48 h. (C) Western blotting analysis of E-cadherin and α-SMA protein expressions in A549 cells with or without IL-17 treatment for 48 h. The histogram shows the average volume density corrected for the loading control (GAPDH). **P<0.01.

The normal A549 cells displayed typical cobblestone-like morphology by inverted microscope, while IL-17-treated A549 cells showed spindle-shaped morphology (Fig 1A). To characterize the morphological changes stimulated by IL-17, we investigated the expression of phenotypic markers in A549 cells by Quantitative RT-PCR analysis. It was found that IL-17-treated A549 cells show decreased expression of E-cadherin—an epithelial marker and increased expression of α-SMA-a mesenchymal marker, compared to control cells (Fig 1B and S1 Fig). In addition, similar results were confirmed by western blotting (Fig 1C). These results suggest that IL-17 initiated A549 cells to undergo EMT.

### IL-17 enhanced TGF-β1 expression

**Fig 2:**
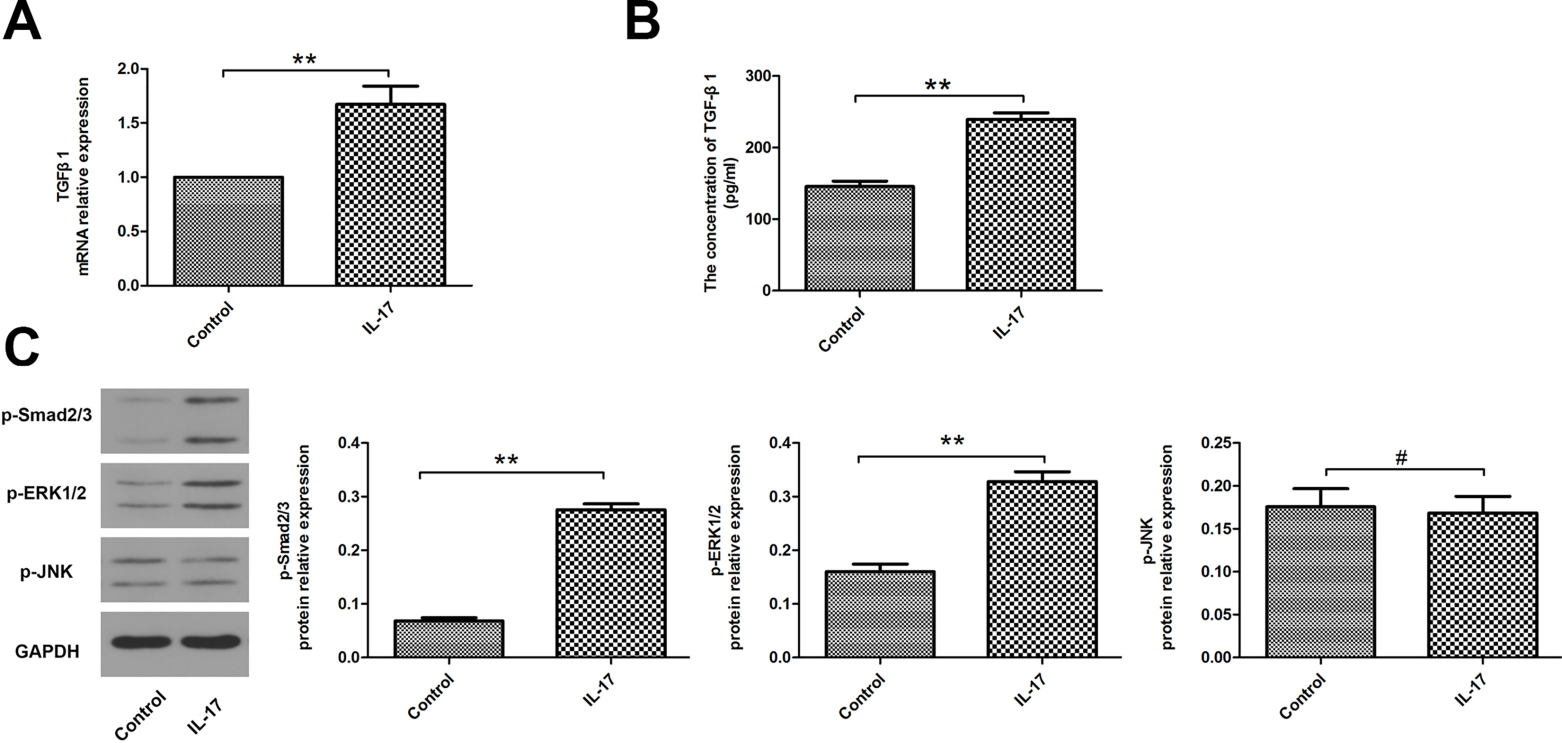
IL-17 enhanced TGF-β1 expression. The A549 cells were stimulated with IL-17 at a final concentration of 100 ng/ml for 48 h. (A) Quantitative RT-PCR analysis of TGF-β1 mRNA expression with or without IL-17 treatment for 48 h. (B) ELISA analysis of TGF-β1 protein expression in the culture medium with or without IL-17 treatment for 48 h. (C) Western blotting analysis of p-Smad2/3, p-ERK1/2 and p-JNK in A549 cells with or without IL-17 treatment for 48 h. The histogram shows the average volume density corrected for the loading control (GAPDH). **P<0.01, # *P*>0.05.

We examined TGF-β1 expression to assess its role in EMT initiated by IL-17. We found that IL-17-treated A549 cells showed up-regulated TGF-β1 expression in mRNA and protein level (Fig 2A and 2B), accompany with increased levels of phosphorylated Smad2/3 and phosphorylated ERK1/2 in protein level. However, there were no significant differences in the expression of phosphorylated JNK in A549 cells with or without IL-17 treatment (Fig 2C). These results suggest that IL-17 can not only increase TGF-β1 expression, but also enhance Smad2/3 and ERK1/2 activity.

### Effects of Smad2/3 and ERK1/2 Inhibitors on IL-17- initiated EMT

**Fig 3:**
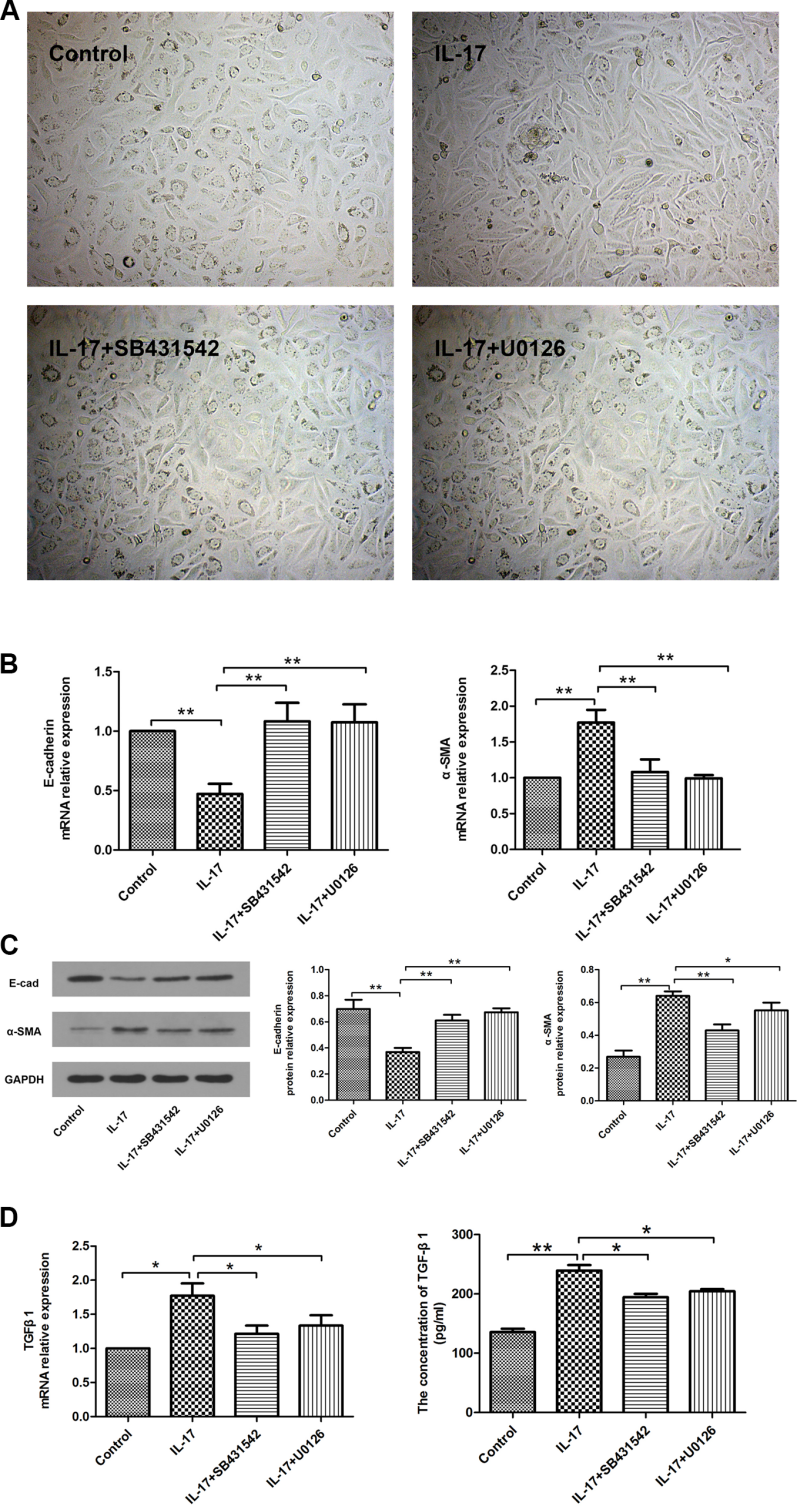
Effects of Smad and ERK inhibitors on IL-17-initiated EMT. SB431542: Smad inhibitor; U0126: ERK inhibitor. (A) Phase contrast analysis (magnification ×200) of morphological changes detected in A549 cells. (B) Quantitative RT-PCR analysis of E-cadherin and α-SMA mRNA expressions. (C) Western blotting analysis of E-cadherin and α-SMA protein expressions in A549 cells. The histogram shows the average volume density corrected for the loading control (GAPDH). (D) Quantitative RT-PCR analysis of TGF-β1 mRNA expression in A549 cells and ELISA analysis of TGF-β1 protein expression in the culture medium. **P<0.01,*P<0.05.

**Fig 4:**
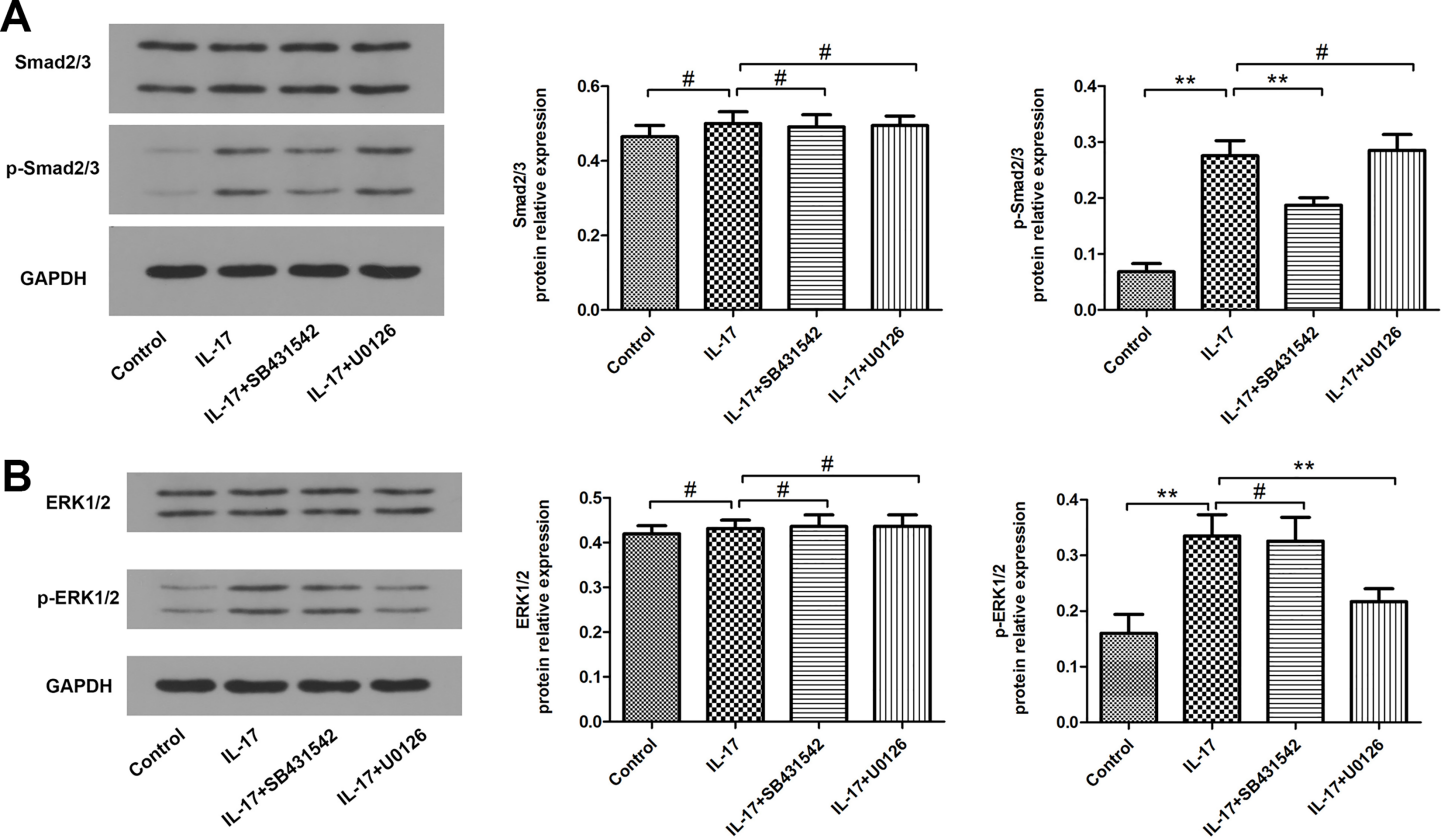
Effects of Smad and ERK inhibitors on Smad and ERK signaling pathways. SB431542: Smad inhibitor; U0126: ERK inhibitor. (A) Western blotting analysis of Smad2/3 and p-Smad2/3 protein expressions in A549 cells. The histogram shows the average volume density corrected for the loading control (GAPDH). (B) Western blotting analysis of ERK1/2 and p-ERK1/2 protein expressions in A549 cells. The histogram shows the average volume density corrected for the loading control (GAPDH). **P<0.01,#*P*>0.05.

In order to elucidate the role of the signaling pathways in IL-17-initiated EMT, we analyzed Smad and ERK pathways with pharmacological inhibitors. SB431542 is a potent and specific inhibitor of TGF superfamily type I activin receptor-Likekinase (ALK) receptors ALK4, ALK5, and ALK7 [14]. As shown in Fig 3D, pre-treatment with SB431542 signifcantly inhibited the enhancement of TGF-β1 mRNA and protein levels. As shown in Fig 4, SB431542-treated A549 cells showed lower Smad2/3 phosphorylation comparable to IL-17-treated A549 cells. However, SB431542 pre-treatment had no effect on the phosphorylation of ERK1/2. Furthermore, A549 cells treated with SB431542 together with IL-17 showed attenuated down-regulation of E-cad expression and up-regulation of α-SMA expression compared with A549 cells treated with IL-17 only (Fig 3). We also assessed ERK signaling pathways which might be involved in IL-17-initiated EMT. Likewise, preincubating A549 cells with U0126, a selective inhibitor of ERK pathway, also correspondingly altered ERK1/2 phosphorylation, TGF-β1, E-cad and α-SMA expression comparable to the IL-17 treatment (Figs 3 and 4). Together, these results demonstrate that both the Smad and ERK pathways are responsible for IL-17-induced EMT.

## Discussion

Pulmonary fibrosis is a chronic and usually progressive lung disease characterized by the accumulation of fibroblasts, myofibroblasts, collagen, and other extracellular matrix proteins in the interstitium of the lung [15]. Myofibroblast is thought to be of significance in the propagation of fibrosis with evolution to terminal end-stage fibrotic lung disease [16]. However, the origin of these myofibroblasts in the airway layer is not entirely clear. Recently, a number of studies have demonstrated that myofibroblast has been thought to derive from injured epithelial cells in the process of EMT [17]. Furthermore, many studies have demonstrated the EMT may paly a critical role in the development and progression of idiopathic pulmonary fibrosis [18, 19].

IL-17 is a newly discovered and proinflammatory cytokine which promotes EMT profiles in lung inflammatory diseases, such as obliterative bronchiolitis [7] and COPD [20]. Additionally, several studies have suggested that high expression of IL-17 is increasingly recognized as a potential key in the progression of fibrosis in many major organs such as heart [21], kidney [22] and liver [23]. Of note, cumulative evidence has suggested that IL-17 is closely associated with EMT in IPF [7, 9]. In human IPF, IL-17 expression is increased in the bronchoalveolar lavage fluid compared to patients without IPF [24]. These findings point to an important role of IL-17 in pulmonary fibrosis. However, little is known regarding the role of IL-17 in the regulation of EMT and the underlying mechanisms. To investigate whether IL-17 can induce EMT in lung epithelial cells, A549 alveolar epithelial cell line were cultured in the presence of IL-17 as a model to simulate IPF. As expected, we observed morphological changes from a cobblestone-like epithelial phenotype to a spindle-like mesenchymal phenotype in A549 cells after IL-17 exposure. Moreover, quantitative RT-PCR and western blotting analyses revealed IL-17 treatment can also change the EMT phenotypic markers in A549 cells, such as down-regulated E-cad expression and up-regulated α-SMA expression compared with controls, which is consistent with the studies conducted in A549 cells [25, 26]. As a result, our data demonstrated that IL-17 can induce EMT in A549 cells. However, the underlying mechanisms of the effects of IL-17 on EMT have not been clearly elucidated. Of note, IL-17 was found to enhance TGF-β1 expression. Meng et al [23] found that IL-17 signaling facilitates production of IL-6, IL-1β, and TNF-α by inflammatory cells, and increases the expression of TGF-β1, the major pro-fibrogenic cytokine. Furthermore, Okamoto et al [27] demonstrated that a skin fibroblast cell line expressed increased TGF-β, CTGF and collagen after the addition of recombinant IL-17 and IL-17 deficiency also attenuated skin thickness in TSK-1 mice. Our results are in accordance with these results that IL-17 exposure can enhance TGF-β1 expression. However, the roles of TGF-β1 signaling pathway in the IL-17-mediated EMT remain to be fully understood.

TGF-β1 is a pleiotropic cytokine that has a crucial role in many aspects of the lung fibrotic response and has long been believed to be a central mediator of this response [28]. Important intracellular mediators of TGFβ signaling are members of the Smad family which have key roles in exerting TGFβ-induced EMT. TGFβ isoforms exert their cellular effects by binding to the TGF-β type II receptor (TβRII), and this binding facilitates activation of TGF-β type I receptor (TβRI) kinase, which leads to the activation of Smad2 and Smad3. Phosphorylated Smads partner with cytosolic Smad4 and form a heteromeric Smad complex, which translocates to the nucleus where they cooperate with other transcription factors, co-activators and co-repressors to regulate the transcription of specific genes [29–31]. Xu et al [32] found that TGF-β1 induced A549 cells EMT through Smad activation. Given the role of the Smad signaling pathway in TGF-β family signaling, IL-17 may induce EMT through TGF-β/Smad signaling pathway. To address this assumption, we conducted studies with A549 cells to assess the impact of expression of phosphorylated Smad2/3 and total Smad2/3 during EMT stimulated by IL-17. Indeed, we found that IL-17 treatment signifcantly increased the phosphorylation of Smad2/3 in A549 cells. However, IL-17 treatment did not affact the expression of total Smad2/3 in A549 cells. It suggested that Smad2/3 pathway involved in the process of EMT stimulated by IL-17. To further elucidate the role of Smad pathway in EMT initiated by IL-17, SB431542, a potent and specific inhibitor of TβRI kinases (ALK-4,-5,-7), was used to inhibit IL-17-induced phosphorylation of Smad2/3 in A549 cells. We found that treatment of A549 cells with SB431542 can inhibit morphological changes and phenotypic markers expression, such as up-regulated E-cad expression and down-regulated α-SMA expression. Taken together, these observations provide compelling evidence to further support our emerging view that IL-17 induces A549 alveolar epithelial cells to undergo EMT via the TGF-β1 mediated Smad2/3 activation.

Additionally, TGF-β1 can also activate non-Smad signaling pathway [33], such as the MAPK signaling cascade including ERK1/2, JNK and p38 kinase. Of note, ERK1/2 signaling pathway has a sophisticated and intimate relationship with the TGF-β1 system in regulating tumorigenesis [34]. Moreover, ERK1/2 activity has been considered to be a prerequisite for TGF-β1-mediated EMT in vitro [35]. Human lung biopsy samples show increased ERK1/2 signaling in IPF samples compared with normal lungs [36]. Madala et al [37]demonstrated that inhibition of the MEK/ERK pathway in vivo attenuated the progression of fibrosis and physiologic alterations in lung mechanics. Therefore, we also conducted studies to explore the role of ERK pathway in the IL-17-mediated EMT. Like the Smad2/3, enhanced ERK phosphorylation can be consistently detected in A549 cells after IL-17 stimulation. Furthermore, treatment of A549 cells with U0126, an ERK1/2 inhibitor, inhibited corresponding morphological changes and phenotypic markers expression in A549 cells. Therefore, our data support that IL-17 enhances ERK1/2 activity, and by which they promote A549 cells EMT during the course of IPF, which is consistent with the studies conducted in bronchial epithelial cells [38]. Finally, these results support the hypothesis that IL-17-mediated EMT may occur via the induction of TGF-β1 and associated signaling pathways, such as Smad2/3 and ERK1/2 signaling pathways.

In conclusion, this study has shown that IL-17 can induce A549 alveolar epithelial cells to undergo EMT via the TGF-β1 mediated Smad2/3 and ERK1/2 activation. This results not only provide new insights into the cellular mechanisms that mediate EMT in human lung epithelial cells, but also bear the potential novel treatment targets that could benefit patients by reducing pulmonary fibrosis and leading to an improved prognosis.

## Supporting information

S1 Fig IL-17 induced EMT in A549 cells. (A) A549 cells were treated with various concentrations IL-17 (0, 10, 25, 50, 100 ng/ml) for 48 h and E-cad mRNA levels were examined by Quantitative RT-PCR (normalized to GAPDH mRNA levels). (B) A549 cells were treated with various concentrations IL-17 (0, 10, 25, 50, 100 ng/ml) for 48 h and α-SMA mRNA levels were examined by Quantitative RT-PCR (normalized to GAPDH mRNA levels). (C) A549 cells were treated without or with 100 ng/ml IL-17 for different periods of time (0, 12, 24, 48 h) and E-cad mRNA levels were examined by Quantitative RT-PCR (normalized to GAPDH mRNA levels). (D) A549 cells were treated without or with 100 ng/ml IL-17 for different periods of time (0, 12, 24, 48 h) and α-SMA mRNA levels were examined by Quantitative RT-PCR (normalized to GAPDH mRNA levels). **P<0.01,#*P*>0.05.
(TIF)
