# Supplementary figures and images for: Interleukin-17 induces human alveolar epithelial to mesenchymal cell transition via the TGF-β1 mediated Smad2/3 and ERK1/2 activation
Source: PLoS One. 2017 Sep 5;12(9):e0183972. doi: 10.1371/journal.pone.0183972 (PMC5584923; doi:10.1371/journal.pone.0183972)

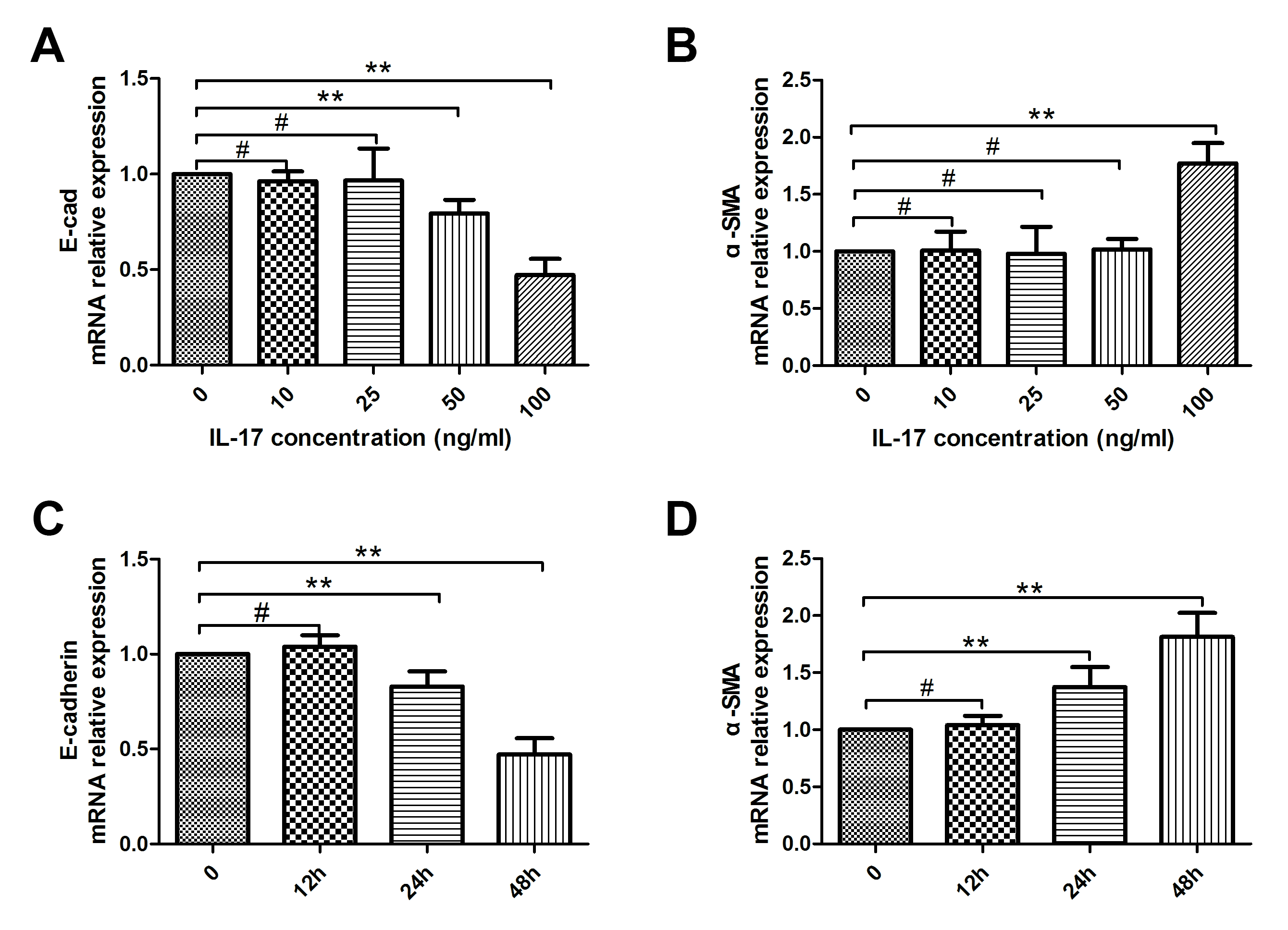

Supplement: S1 Fig — (A) A549 cells were treated with various concentrations IL-17 (0, 10, 25, 50, 100 ng/ml) for 48 h and E-cad mRNA levels were examined by Quantitative RT-PCR (normalized to GAPDH mRNA levels). (B) A549 cells were treated with various concentrations IL-17 (0, 10, 25, 50, 100 ng/ml) for 48 h and α-SMA mRNA levels were examined by Quantitative RT-PCR (normalized to GAPDH mRNA levels). (C) A549 cells were treated without or with 100 ng/ml IL-17 for different periods of time (0, 12, 24, 48 h) and E-cad mRNA levels were examined by Quantitative RT-PCR (normalized to GAPDH mRNA levels). (D) A549 cells were treated without or with 100 ng/ml IL-17 for different periods of time (0, 12, 24, 48 h) and α-SMA mRNA levels were examined by Quantitative RT-PCR (normalized to GAPDH mRNA levels). **P<0.01,#P>0.05. (TIF) [file pone.0183972.s001.tif]
